# A Comparison of T_2_ Relaxation-Based MRI Stroke Timing Methods in Hyperacute Ischemic Stroke Patients: A Pilot Study

**DOI:** 10.1177/1179573520943314

**Published:** 2020-09-12

**Authors:** Bryony L McGarry, Robin A Damion, Isabel Chew, Michael J Knight, George WJ Harston, Davide Carone, Peter Jezzard, Amith Sitaram, Keith W Muir, Philip Clatworthy, Risto A Kauppinen

**Affiliations:** 1 School of Psychological Science, University of Bristol, Bristol, UK; 2 Acute Stroke Programme, Radcliffe Department of Medicine, University of Oxford, Oxford, UK; 3 Institute of Neuroscience and Psychology, Queen Elizabeth University Hospital, University of Glasgow, Glasgow, UK; 4 Stroke Neurology, Southmead Hospital, North Bristol NHS Trust, Bristol, UK; 5 Faculty of Engineering, University of Bristol, Bristol, UK

**Keywords:** Ischemic stroke, unknown onset, T_2_ relaxation, signal intensities, DWI/FLAIR mismatch

## Abstract

**Background:**

T_2_ relaxation-based magnetic resonance imaging (MRI) signals may provide onset time for acute ischemic strokes with an unknown onset. The ability of visual and quantitative MRI-based methods in a cohort of hyperacute ischemic stroke patients was studied.

**Methods:**

A total of 35 patients underwent 3T (3 Tesla) MRI (<9-hour symptom onset). Diffusion-weighted (DWI), apparent diffusion coefficient (ADC), T_1_-weighted (T_1_w), T_2_-weighted (T_2_w), and T_2_ relaxation time (T_2_) images were acquired. T_2_-weighted fluid attenuation inversion recovery (FLAIR) images were acquired for 17 of these patients. Image intensity ratios of the average intensities in ischemic and non-ischemic reference regions were calculated for ADC, DWI, T_2_w, T_2_ relaxation, and FLAIR images, and optimal image intensity ratio cut-offs were determined. DWI and FLAIR images were assessed visually for DWI/FLAIR mismatch.

**Results:**

The T_2_ relaxation time image intensity ratio was the only parameter with significant correlation with stroke duration (*r* = 0.49, *P* = .003), an area under the receiver operating characteristic curve (AUC = 0.77, *P* < .0001), and an optimal cut-off (T_2_ ratio = 1.072) that accurately identified patients within the 4.5-hour thrombolysis treatment window with sensitivity of 0.74 and specificity of 0.74. In the patients with the additional FLAIR, areas under the precision-recall-gain curve (AUPRG) and F_1_ scores showed that the T_2_ relaxation time ratio (AUPRG = 0.60, F_1_ = 0.73) performed considerably better than the FLAIR ratio (AUPRG = 0.39, F_1_ = 0.57) and the visual DWI/FLAIR mismatch (F_1_ = 0.25).

**Conclusions:**

Quantitative T_2_ relaxation time is the preferred MRI parameter in the assessment of patients with unknown onset for treatment stratification.

## Introduction

Unknown time of symptom onset due to “wake-up stroke” or lack of witness is a common contraindication for reperfusion therapies for ischemic stroke.^1,2^ Intra-arterial administration of thrombolytic agents is considered safe within 6 hours of onset but is not routine,^3^ and patients with large vessel occlusion (LVO) can now be considered for mechanical thrombectomy if symptom onset was within the last 24 hours.^4,5^ For the many patients without LVO, intravenous (IV) thrombolysis using recombinant tissue plasminogen activator (rtPA) is the only alternative.^5^ Current guidelines dictate rtPA must be administered within 4.5 hours from symptom onset due to increased risk of hemorrhage after this time point.^5^ Multiparametric magnetic resonance imaging (MRI) reveals pathophysiological changes in the ischemic brain parenchyma, enabling diagnosis, insight into the extent of ongoing tissue damage, and inference of stroke duration.^6^ MRI may, therefore, aid treatment stratification of ischemic stroke patients with unknown symptom onset time by identifying patients who (1) are likely to be within the 4.5-hour IV rtPA treatment window or (2) have sufficient viable tissue that would suggest they may benefit from reperfusion therapy regardless of onset time.^7,8^ This study focused on imaging methods aimed at achieving (1).

MRI contrasts, including diffusion-weighted imaging (DWI) or the quantitative measure of diffusion, the apparent diffusion coefficient (ADC), are extremely sensitive to ischemia. Studies using rodent models showed DWI signals increase and ADC values decrease sharply within minutes of ischemia onset at the same cerebral blood flow (CBF) threshold for catastrophic energy failure.^9,10^ Regions with low ADC, therefore, reflect tissue undergoing cytotoxic edema and the associated cellular changes.^6,11^ The wide dynamic range of ADC provides an excellent contrast to the non-ischemic brain, and so is useful for diagnosing ischemia and localizing affected brain tissue.^6,12^ However, ADC values in patients remain consistently low for several days after the insult,^13^ making it an unsuitable parameter for stroke timing. Preclinical studies have shown that the T_1_ and T_2_ relaxation times that contribute to the signal of weighted images typically acquired in the clinic, such as DWI, T_2_-weighted (T_2_w), and T_2_w FLAIR (FLAIR), also change early during ischemia but, compared with ADC, changes are small.^6^ T_1_ and T_2_ relaxation times have been shown to have linear time dependency in rat stroke models,^14,15^ and for T_2_, this has been translated to human stroke,^16-19^ suggesting quantification of the T_2_ relaxation time may be a suitable method for stroke timing.

The T_2_ relaxation-based signal changes that occur in DWI and FLAIR images after stroke have also been exploited for stroke timing using visual^20-22^ and quantitative methods.^23,24^ For both approaches, if the signal in the ischemic region in the FLAIR image (identified by DWI or ADC) is deemed not to be hyperintense, it is likely that the patient is within the treatment window and thus eligible for rtPA. For the visual DWI/FLAIR mismatch approach, the presence of a “mismatch” where a lesion is visible on DWI but not FLAIR scans, indicates patient eligibility.^20-22^ For the quantitative approach, the eligibility is determined by whether the ratio of image intensity values between ischemic and nonischemic reference regions is below a specific optimal cut-off.^23,24^ This approach has been studied using image intensities from ADC, DWI, T_2_w, FLAIR, and T_2_ relaxation images in animal models of ischemia^25,26^ and ischemic stroke patients,^19,23,24,27,28^ but the overall performance of these parameters has not been directly compared in hyperacute stroke patients.

The recent results of the “WAKE-UP” stroke trial^29^ in which patients with DWI/FLAIR mismatch treated with IV rtPA showed an 11.5% increased favorable outcome compared with placebo and have provided further impetus for investigating the clinical benefit and application of MRI for timing the ischemic stroke. Potential stroke timing methods must be able to successfully discriminate between patients within and beyond the 4.5-hour rtPA time window. High sensitivity is essential to identify as many patients as possible who are eligible for rtPA, and high specificity is also imperative to avoid potentially harmful treatment. In this pilot study, the performance of quantitative and visual MRI-based stroke timing methods derived from the same cohort of hyperacute ischemic stroke patients was compared. The focus was on image intensity ratios of ADC, DWI, T_2_w, T_2_ relaxation and FLAIR images, and the DWI/FLAIR mismatch.

## Methods

### Patients

Patients were recruited from North Bristol NHS Trust Frenchay and Southmead Hospitals (Bristol), Queen Elizabeth University Hospital (Glasgow), and University of Oxford Radcliffe Department of Medicine’s Acute Vascular Imaging Centre (Oxford) (between 03/2014 and 08/2018). Time of witnessed symptom onset, National Institute Health Stroke Scale (NIHSS), and the attending physician’s stroke classification according to the Oxford Community Stroke Project Classification^30^ were recorded on admission. All stroke subtypes were considered for enrolment, including lacunar stroke (LACS), partial anterior stroke (PACS), posterior circulation stroke (POCS), and total anterior circulation stroke (TACS). Before enrolment, all patients received noncontrast computerized tomography (NCCT) scans and were treated according to the standard-of-care protocol, including administration of IV rtPA if eligible. Patients were not offered endovascular reperfusion therapy at any site.

Patients, or their legal representative, provided informed consent. Enrolled patients had MRI scans within 9 hours of symptom onset. Exclusion criteria after enrolment included withdrawal from involvement in the study after initial consent, early termination of scan due to claustrophobia, unclear diagnosis, uncertainty regarding symptom onset time, movement artefacts, error in scan protocol, no evident lesion on ADC images, evidence of bilateral stroke, bias field problems in weighted images, and presence of both periventricular and parenchymal white matter hyperintensities on T_2_w images.

The study received ethical approval from the South West Frenchay Research Ethics Committee (ref 13/SW/0256), Scotland A REC (ref 16/SS/0223), and UK National Research Ethics Service committees (refs 12/SC/0292 and 13/SC/0362) for participants in Bristol, Oxford, and Glasgow, respectively. Ethical approval allowed patients to be in the scanner for up to 20 minutes at Bristol and up to 30 minutes at Oxford and Glasgow. Total scan time allowed included set up of the patient and localizers as well as the MRI protocol. The study was carried out in accordance with the Declaration of Helsinki.

### MRI

All sites used a 3T (3 Tesla) MRI scanner with a 32-channel head coil (Bristol: Philips Achieva, Glasgow: Siemens Magnetom Prisma, Oxford: Siemens Magnetom Verio). The MRI protocol detailed in Table 1 included multi *b*-value diffusion for computation of DWI and ADC images, multi-echo T_2_ for computation of T_2_ relaxation and T_2_w images, and 3D (3-dimensional) T_1_w images for anatomical reference and registration. The approved scan time at Oxford and Glasgow enabled the acquisition of T_2_w FLAIR as well.

**Table 1.** MRI acquisition parameters.

	Sequence	TR (ms)	TE (ms)	Resolution (mm^3^)			Acquisition time (min: s)
Multi-echo T_2_							
Bristol	GRASE	3000	20, 40, 60, 80, 100	0.6 × 0.6 × 2.3	–	–	3:09
Glasgow	TSE	12 500	9.5, 66, 123	1.7 × 1.7 × 2.0	–	–	2:03
Oxford	TSE	12 000	7.7, 77, 177	1.8 × 1.7 × 2.0	–	–	1:50
					TI (ms)	Flip Angle	
3D T_1_w							
Bristol	FFE	6.84	3.18	1.0 × 1.0 × 1.1	–	8°	4.54
Glasgow	MP RAGE	2200	2.28	0.9 × 0.9 × 0.9	900	9°	5.07
Oxford	MP RAGE	1800	4.55	1.5 × 1.5 × 1.0	900	8°	2.06
T_2_w FLAIR							
Glasgow	TSE	10 000	93	0.9 × 0.9 × 5.0	2500	150°	3.02
Oxford	TSE	9000	96	1.9 × 1.9 × 2.0	2500	150°	2.08
					*b* value, s/mm^2^ (multiplicity)	Independent gradient directions	
Diffusion							
Bristol	SE-EPI	3009	60.5	1.2 × 1.2 × 4.4	0(1), 1000(3)	3	0.37
Glasgow	SE-EPI	8000	90	0.9 × 0.9 × 2.0	0(3), 1000(20)	20	3.03
Oxford	SE-EPI	5300	91	1.8 × 1.8 × 5.0	0(1), 1000(1)	3	1.00

Abbreviations: GRASE, Gradient and Spin Echo; mm, millimeters; MP RAGE, 3D T_1_w Magnetization Prepared Rapid Acquisition Gradient Echo; ms, milliseconds; s, seconds; SE-EPI, Spin-Echo Echo Planar Imaging; TE, time of echo; TR, time of repetition; TI, time of inversion; TSE, Turbo Spin Echo; T_1_ FFE, radiofrequency spoiled incoherent gradient echo; T_2_w FLAIR, slice-selective T_2_w Fluid Attenuated Inversion Recovery.

### Image processing and analysis

**Figure 1.**
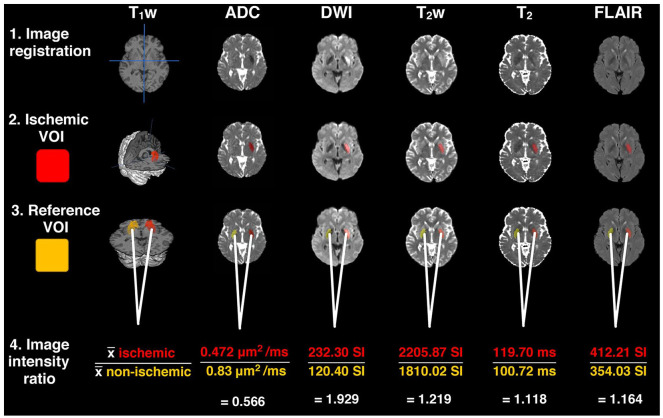
Summary of image processing and steps. (1) All images were registered to the same space. (2) ADC and T_2_ limits defined the ischemic VOI. (3) Nonischemic reference VOIs were created by reflecting the ischemic VOI across the vertical axis, applying ADC and T_2_ limits and manually editing if necessary. (4) Image intensity ratios were determined by dividing the average image intensity of the ischemic VOI by the average image intensity in the nonischemic VOI. Images shown are from the same patient (time from onset = 6 h 49 min, age = 59, thrombolysed before MRI, NIHSS = 13) and are representative of the image quality of scans acquired for all patients. ADC indicates apparent diffusion coefficient; VOI, volumes of interest.

Image processing and analysis steps are illustrated in Figure 1 and described in detail below. These methods were developed and implemented by Knight et al^19^ and Damion et al,^18^ and programs used included MATLAB release 2016b (The MathWorks, Inc, Natick, MA), FSL (FMRIB, Oxford, UK), MANGO version 4.1 (Research Imaging Institute, UT Health Science Center, San Antonio, TX) and SPM12 (Wellcome Trust Centre for Neuroimaging).

#### Image computation

For diffusion data using 3 orthogonal diffusion-sensitizing gradients at a common *b* value, orientation independent ADC images were calculated using

(1) $$\mathrm{ADC}=\frac{-\ln\left(\frac{S_{1}S_{2}S_{3}}{S_{0}^{3}}\right)}{3b},$$

where $S_{0}$ is the signal without diffusion weighting, b is the *b* value, and $S_{1}$, $S_{2}$, and $S_{3}$ are the signal intensities at the 3 orthogonal directions.

For diffusion-weighted data with 20 independent diffusion-gradient directions (*b* = 1000 mm^2^ s^−1^) and 3 *b* = 0 images, ADC values were obtained from mean diffusivity maps, which were computed using FSL DTIFIT. Effective diffusion-weighted images were created for *b* = 1000 mm^2^/s using the registered *S*_0_ and ADC images via

(2) $$\mathrm{DWI}=S_{0}e^{\left(-1000\;\times \;ADC\right)}.$$

Echo-summed T_2_w images were computed by summing all echoes of the TE series, followed by bias correction using FSL FAST.^31^ Calculation of T_2_ relaxation images involved fitting a mono-exponential decay on a voxel-wise basis.

#### Image registration

To ensure correct alignment between voxels across all images and that the midline of the brain was consistently defined, for each patient, T_1_w, ADC, DWI, T_2_w, T_2_ relaxation, and FLAIR images were brain extracted using FSL BET^32^ and registered to the same space. This involved nonlinear registration of the ADC images to the T_2_w image space using FSL FNIRT via the diffusion S_0_ image and the echo summed T_2_w image. If T_1_w images were available, the T_2_w image space was linearly registered (6 degrees of freedom) to the T_1_w image space, which was subsequently registered to the MNI frame at 1 mm^3^ isotropic resolution using FSL FLIRT.^33^ Where T_1_w images were not available, the T_2_w image space was, instead, directly registered (linearly) to the MNI frame at 1 mm^3^ isotropic resolution. All images were then linearly registered to the MNI frame using the registration maps (or combination of) generated by previous stages. The quality of co-registration across all image types was assessed visually by 2 independent assessors using SPM12 (BLM & IC). All images were deemed to be acceptably co-registered.

#### Lesion identification

The definition of ischemic voxels was those with ADC values >0.2-0.4 and <0.55-0.6 μm^2^/ms as well as ADC values less than one half-width half-maximum from the median ADC of nonischemic tissue. Limits of T_2_ >30 and <200 ms were also applied to reduce the contribution from cerebral spinal fluid (CSF). These criteria were used to create lesion masks to define ischemic volumes of interest (VOIs). Refinement of ischemic VOIs involved removing all but the largest cluster (or clusters if there was more than 1 lesion). Selection of nonischemic reference VOIs required reflecting the ischemic VOI about the midline of axial slices (medial/lateral) applying the above T_2_ limits and manually editing if CSF was visible within the VOI.

#### Computation of image intensity ratios

To approximate changes in image intensities due to ischemia, image intensity ratios were calculated for each patient and image type, which involved dividing the mean value in the ischemic VOI by the mean value in the nonischemic reference VOI. Pre-ratio values were in milliseconds (ms) for T_2_ relaxation images, µm^2^/ms for ADC, and signal intensities (SI, arbitrary units) for weighted images (DWI, T_2_w, FLAIR). The purpose of computing ratios instead of using hemispheric differences in mean values was to reduce concerns that may arise from differences in pulse sequences, structures, and parameters between imaging sites.

#### Visual DWI/FLAIR mismatch

Four independent raters with experience in stroke MRI (RD, PC, AS, RAK) assessed DWI and FLAIR images. Raters were blinded to clinical details and asked to identify cases showing a “match” (visible hyperintensities in the same region on DWI and FLAIR), a “mismatch” (visible hyperintensity on DWI, not FLAIR), or cases they were uncertain about (no response). Raters viewed and applied thresholds to images according to personal preference. An arbitrator (KWM) with more than 25 years of experience in stroke MRI assessed images where there was uncertainty or disagreement between raters. Final classification as “match” or “mismatch” was based on majority classification, or KWM’s adjudication when there was a 2-2 split in opinion.

### Statistical analysis

Data from all 35 patients and the subcohort with the additional FLAIR scans were analyzed separately. This separation was to allow for a fair comparison of the performance of FLAIR with other MRI classifiers. Statistical analysis was carried out using GraphPad Prism version 8.02 (GraphPad Software, La Jolla, CA), MedCalc Statistical Software version 19.0.5 (MedCalc Software bvba, Ostend, Belgium), and MATLAB release 2019a (The MathWorks, Inc, Natick, MA).

Data were assessed for normality using the Shapiro-Wilk test.^34^ The difference between the average ADC values in the ischemic VOI between thrombolysed and non-thrombolysed patients was assessed using unpaired *t* tests. The relationships of image intensity ratios with time from symptom onset were assessed using Pearson correlations for normally distributed data and Spearman and Kendall correlations for non-normal data. Randolph free-marginal Fleiss Kappa calculator^35,36^ was used to measure the agreement between the 4 DWI/FLAIR mismatch raters. All statistical tests were 2-tailed with a significance level of *P* < .05, and 95% confidence intervals (CIs) were calculated as ±1.96 × the standard error (SE) unless otherwise stated. The performance of MRI classifiers was compared using measures of accuracy (accuracy, sensitivity/recall, specificity), correctness (positive and negative predictive values), and probability (logistic regression). See ITEM 1 in the supplementary material for definitions and explanations of metrics used.

#### All patients

For a visual indication of the overall performance of MRI classifiers, receiver operating characteristic (ROC) curves were plotted. For a numerical indication of overall performance, areas under the ROC curves (AUC) were calculated and statistically compared using non-parametric methods that control for multiple comparisons.^37^ Optimal image intensity ratio cut-offs were derived by the maximum Youden J Index, which identifies the cut-off that minimizes misclassification by giving equal weight to sensitivity and specificity.^38^ Accuracy, sensitivity, specificity, positive predictive value (PPV), and negative predictive value (NPV) associated with these cut-offs were also calculated.

For further insight into the predictive performance of MRI classifiers, we also performed logistic regression analyses using MATLAB’s Classification Learner app. Predictive generalized linear models were produced for individual image intensity ratios as well as a combination of all image intensity ratios. Predictive generalized linear models took the form of

(3) $$Y=\alpha +\beta_{1}\chi_{1}+\beta_{2}\chi_{2}+\beta_{3}\chi_{3}+\beta_{4}\chi_{4}\ldots ,$$

where Y is the predicted log odds of the patient being within the treatment window (Y = 1 for onset time <270 minutes, Y = 0 for onset time >270 minutes), α is the estimated intercept, β_1...n_ are the estimated regression coefficients, and χ_1. . .n_ are the predictors (eg, image intensity ratio of ADC, DWI, T_2_w, T_2_).^36^ The probability of being within the treatment window (*P*) could thus be calculated as

(4) $$P=\frac{e^{\left(\alpha +\;\;\beta_{1}\chi_{1}+\;\;\beta_{2}\chi_{2}+\;\;\beta_{3}\chi_{3}+\;\beta_{4}\chi_{4}\ldots \right)}}{1+e^{\left(\alpha +\;\;\beta_{1}\chi_{1}+\;\;\beta_{2}\chi_{2}+\;\;\beta_{3}\chi_{3}+\;\beta_{4}\chi_{4}\ldots \right)}}.$$

Equation (4) was used to generate probability plots for individual parameters providing a visual indication of the predictive power of models. The overall performance of the models was evaluated by comparing the significance of the χ^2^ statistic and comparing the Akaike information criterion (AIC) values^37^ corrected for sample size (AIC_c_, see supplementary materials ITEM 1), where a low AIC_c_ indicates a high-quality model.^39^ For the combined model, the significance level of the *t*-statistic associated with each of the estimated coefficients (β) was used to assess the extent of the contribution of each parameter to the model.

#### Subcohort with FLAIR MRI

Different methods of evaluation were applied to the subcohort of patients with the additional FLAIR scans due to the imbalance in class sizes (n = 5 with onset times less than 4.5 hours, n = 12 with onset times greater than 4.5 hours). In data sets where there is a class imbalance, traditional ROC curves and AUCs are not recommended as they place more weight on the larger class and portray an overly optimistic view of overall performance.^40,41^ Instead, in cases where there is low prevalence in the positive compared with the negative class (ie, 5 patients within the treatment window in this study), it has been recommended to use performance metrics such as precision/PPV and recall/sensitivity, which do not use the true negative contingency class in their definitions.^41-43^ We, therefore, applied the precision-recall-gain (PRG) approach,^42^ which performs well in class-imbalanced data sets while maintaining the benefits associated with ROC analysis (see ITEM 1 in supplementary material).

For a visual indication of the overall performance of MRI classifiers, precision-recall-gain (PRG) curves were plotted, and for a numerical indication, areas under the PRG curves (AUPRG) were calculated using open-source MATLAB software (see http://people.cs.bris.ac.uk/~flach/PRGcurves//). An AUPRG of 0 indicates a trivial (random) classifier, and positive and negative AUPRGs indicate more and less optimal classifiers, respectively. To compare the performance of image intensity ratios with the visual DWI/FLAIR mismatch, F_1_ scores^44^ were calculated for the DWI/FLAIR mismatch and image intensity ratio cut-offs. The F_1_ score gives equal weighting to the importance of precision and recall and is considered an appropriate evaluation measure for imbalanced data sets.^45^ For each parameter, the image intensity ratio with the highest F_1_ score was chosen for comparison.

## Results

Sixty-five patients were enrolled in the study. Thirty were not included in the final analysis due to bilateral stroke (n = 4) and the presence of both periventricular and parenchymal white matter hyperintensities (n = 2), no evidence for ischemia on ADC images (n = 11), uncertainty over stroke diagnosis (n = 3), movement artefacts (n = 1), ADC or T_2_ not acquired (n = 4), vague onset time recorded (n = 3), MRI declined after consent (n = 1), and the early termination of scanning due to claustrophobia (n = 1).

A summary of the clinical and imaging characteristics of all 35 patients and the subcohort with FLAIR scans is shown in Table 2. Total MRI acquisition times were 8 minutes, 14 minutes 9 seconds, and 7 minutes 9 seconds for Bristol, Glasgow, and Oxford, respectively. Results for Shapiro-Wilk tests, and Pearson, Spearman, and Kendall correlations are given in SI Table 1 in the supplementary materials document. Mean ADC values (μm^2^/ms) in ischemic and non-ischemic reference VOIs were consistent with previous reports in patients.^46^ There was no difference between average ADC values in ischemic VOIs between thrombolysed and non-thrombolysed patients, for all 35 patients, thrombolysed: M = 0.50, SD = 0.05 vs non-thrombolysed: M = 0.54, SD = 0.05, *t*(33) = 2.02, *P* = .052, and the subcohort with FLAIR scans, thrombolysed: M = 0.49, SD = 0.06 vs nonthrombolysed: M = 0.54, SD = 0.02, *t*(15) = 1.35, *P* = .197.

**Table 2.** Clinical and imaging characteristics.

	All patients	Subcohort with FLAIR
Patients, No. (%)	35	17
Female, No. (%)	13 (37.1)	3 (17.6)
Age, median (min-max)	68 (31-85)	67 (49-85)
NIHSS,^a^ median (min-max)	8 (1-28)	8 (1-28)
Study site, No. (%)		
Bristol	17 (48.6)	0
Oxford	5 (14.3)	4 (23.5)
Glasgow	13 (37.1)	13 (75.5)
Stroke type,^b^ No. (%)		
LACS	12 (34.3)	6 (35.3)
PACS	12 (34.3)	9 (52.9)
POCS	3 (8.6)	0
TACS	8 (22.9)	2 (11.8)
Left hemisphere, No. (%)	16 (45.7)	9 (52.9)
Thrombolysis		
Patients received rtPA, No. (%)	26 (74.3)	14 (82.4)
Median time from onset to rtPA, hours:mins (min-max)	2:05 (1:02-3:55)	2:32 (1:05-3:55)
Median time from rtPA to MRI, hours:mins (min-max)	3:37 (0:29-7:18)	4:20 (1:10-7:18)
Time from onset to MRI		
All patients, median hours:mins (min-max)	5:34 (2:25-9:29)	6:46 (2:28-9:29)
0-⩽4.5 h		
Patients, No. (%)	16 (45.7)	5 (29.4)
Median hours:mins (min-max)	3:22 (2:25-4:25)	3:00 (2:28-3:10)
>4.5-⩽9.5 h		
Patients, No. (%)	19 (54.3)	12 (70.6)
Median hours:mins (min-max)	6:55 (5:08-9:29)	6:59 (5:34-9:29)
VOI characteristics		
Median ADC lesion volume, mL (min-max)	1.99 (0.04-54.6)	5.50 (0.11-25.01)
Median ischemic ADC, µm^2^/ms (min-max)	0.53 (0.39-0.58)	0.51 (0.39-0.58)
Median nonischemic ADC, µm^2^/ms (min-max)	0.77 (0.36-1.00)	0.79 (0.69-0.89)

Abbreviations: ADC, apparent diffusion coefficient; LACS, lacunar; mL, milliliters; ms, milliseconds; MRI, magnetic resonance imaging; NIHSS, National Institutes of Health Stroke Scale; PACS, Partial Anterior Circulation; POCS, Posterior Circulation; rtPA, recombinant tissue plasminogen activator; TACS, Total Anterior Circulation; VOI, volume of interest.

a Scores on the NIHSS range from 0-42, higher scores indicate greater deficit.

b Strokes classified according to the Oxford Stroke Classification Scale.^30^

### All 35 patients

**Figure 2.**
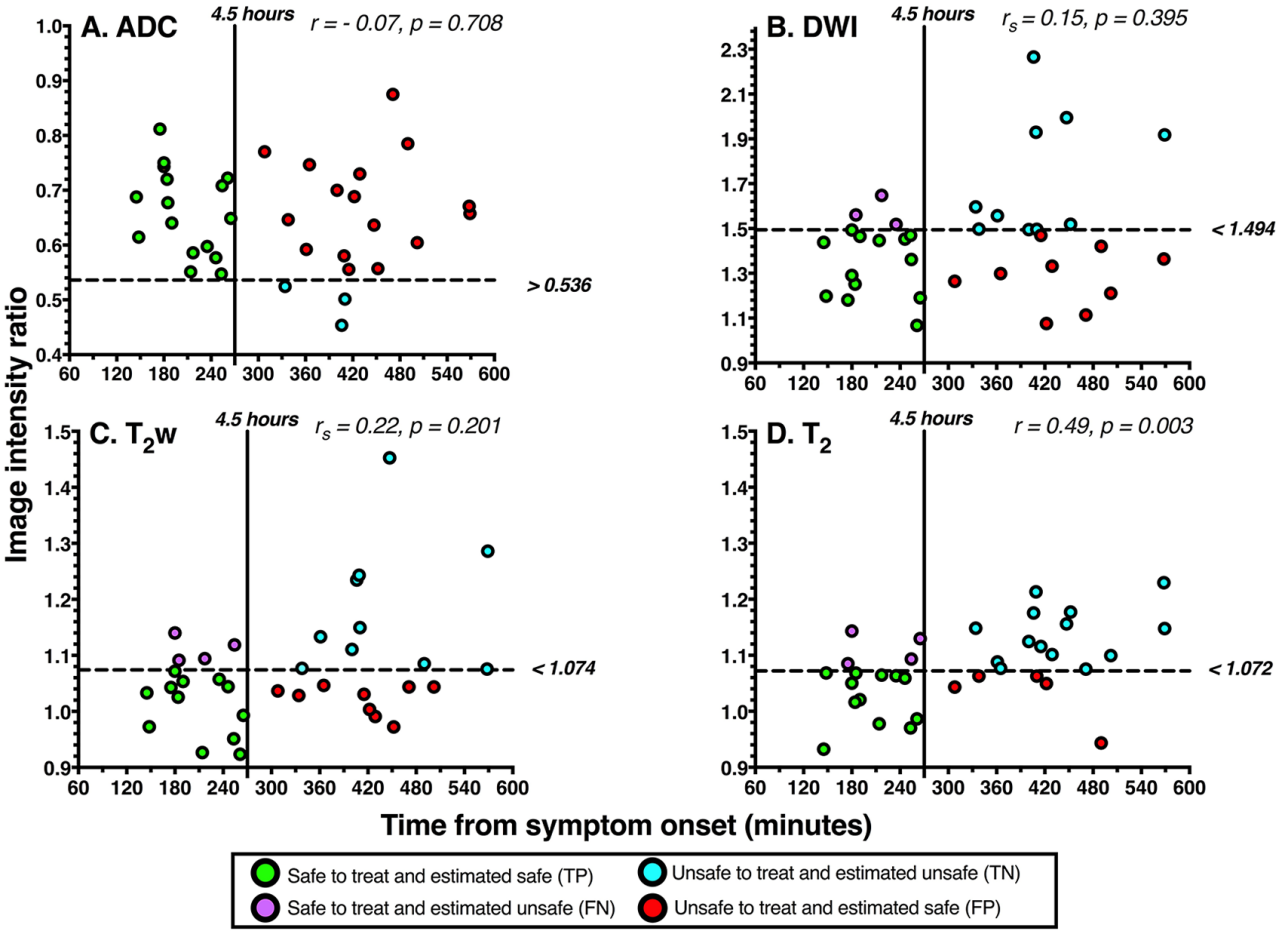
Relationship of image intensity ratios with time from symptom onset for (A) ADC, (B) DWI, (C) T_2_w, and (D) the T_2_ relaxation time. Correlation coefficients and *P* values are shown for each parameter. Pearson correlation coefficients (*r*) are shown for ADC and T_2_ as they were normally distributed. Spearman rank correlation coefficients (*r_s_*) are shown for DWI and T_2_w as they were not normally distributed. Horizontal dashed lines represent optimal image intensity ratio cut-offs identified by the maximum Youden J index, which are labeled to the right of each figure. Vertical solid lines represent the 4.5-hour thrombolysis treatment-window cut-off. Data points represent individual patients and are color-coded according to the classification instructed by the optimal image intensity ratios. Green indicates a true positive case (TP), blue indicates a true negative case (TN), purple indicates a false negative case (FN), and red indicates a false positive case (FP). Data are from all 35 patients. ADC indicates apparent diffusion coefficient; DWI, diffusion-weighted imaging.

Figure 2 shows the T_2_ relaxation time intensity ratio was the only parameter that correlated significantly with time from symptom onset (statistics given in SI Table 1 of supplementary materials).

**Figure 3.**
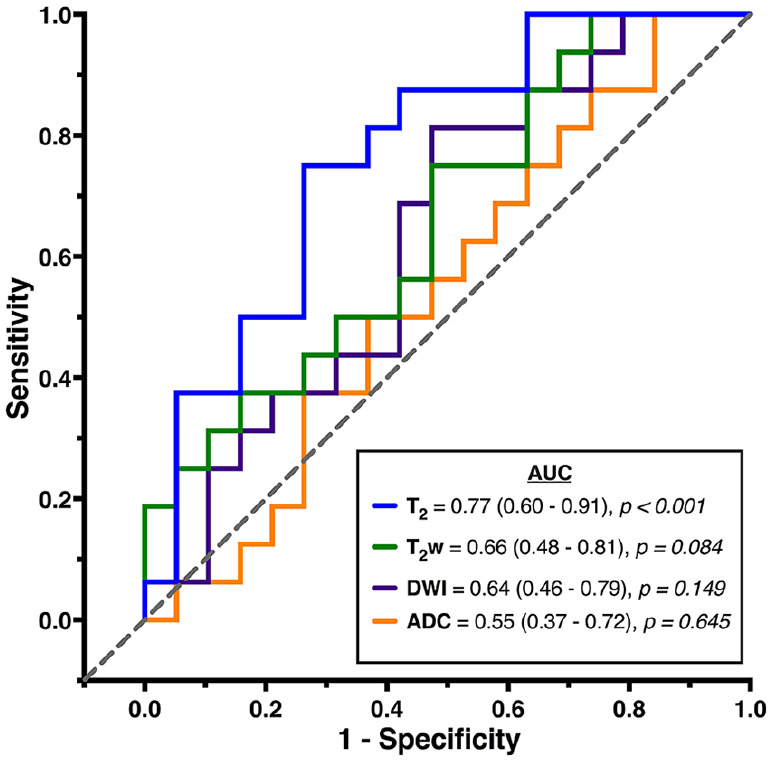
ROC curves showing the overall ability of image intensity ratios for distinguishing between ischemic stroke patients scanned before or after 4.5 hours from symptom onset. Lines closer to the grey dashed 0.5 reference line indicate parameters with poor overall ability. The closer the line to the top left-hand corner of the ROC graph, the higher the overall ability. AUC and *P* values are displayed for each MRI parameter with 95% binomial exact confidence intervals in brackets. An AUC with *P* > .05 indicates an AUC that does not significantly differ from an AUC of 0.5 and therefore performs no better than chance. Results are from all 35 patients. ADC indicates apparent diffusion coefficient; AUC, area under the curve; DWI, diffusion-weighted imaging; MRI, magnetic resonance imaging; ROC, receiver operating characteristic.

The T_2_ relaxation time image intensity ratio also showed the highest overall ability at distinguishing between patients scanned before and after 4.5 hours as the ROC curve was closer to the top left-hand corner of the ROC graph, and it was the only parameter with a significantly high AUC (Figure 3). There was no statistical difference between the AUCs of any of the parameters (see SI Table 2 in supplementary materials).

**Figure 4.**
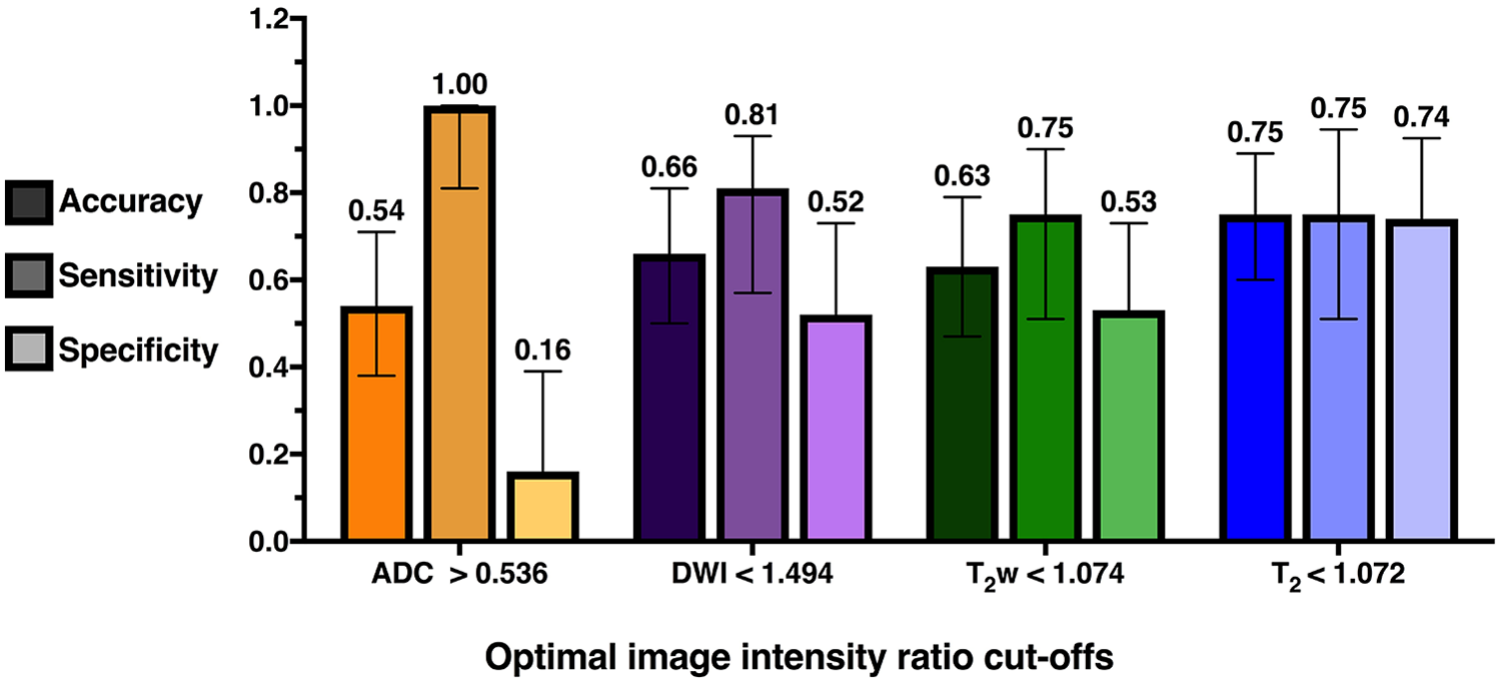
Accuracy, sensitivity, and specificity of optimal image intensity ratio cut-offs identified by the maximum Youden J index. For each parameter, the first darker shaded bar represents the accuracy, the second lighter shaded bar represents sensitivity, and the lightest third bar represents specificity. Corresponding values are labeled above the error bars, which represent the 95% confidence intervals. Results are from all 35 patients. ADC indicates apparent diffusion coefficient; DWI, diffusion-weighted imaging.

Figure 4 shows accuracy, sensitivity, and specificity values for optimal image intensity ratio cut-offs. As seen, the T_2_ relaxation time ratio had the highest accuracy, and the sensitivity and specificity levels were high and comparable. For ADC, DWI, and T_2_w, there was a trade-off, where most patients within the thrombolysis time window were correctly identified (high sensitivity) but at the expense of falsely regarding many patients beyond the time window as within it (low specificity). With these optimal image intensity ratio cut-offs, PPVs were calculated for T_2_ (70.59% CI: 51.79-84.28), ADC (50.0% CI: 45.15-54.85), DWI (59.09% CI: 45.97-71.03), and T_2_w (57.14% CI: 43.43-69.84), and indicate that the T_2_ ratio has the highest probability of correctly predicting whether a patient who is identified by this parameter as being within the treatment window, is actually within the window. Except for ADC, where NPV = 100%, NPVs were comparable for DWI (76.92% CI = 52.46-86.6), T_2_w (71.43% CI = 49.16-86.6), and T_2_ (77.78% CI = 58.97-89.50). The performance of image intensity ratio cut-offs is further illustrated in Figure 2, which shows how patients were classified when cut-offs were applied.

**Figure 5.**
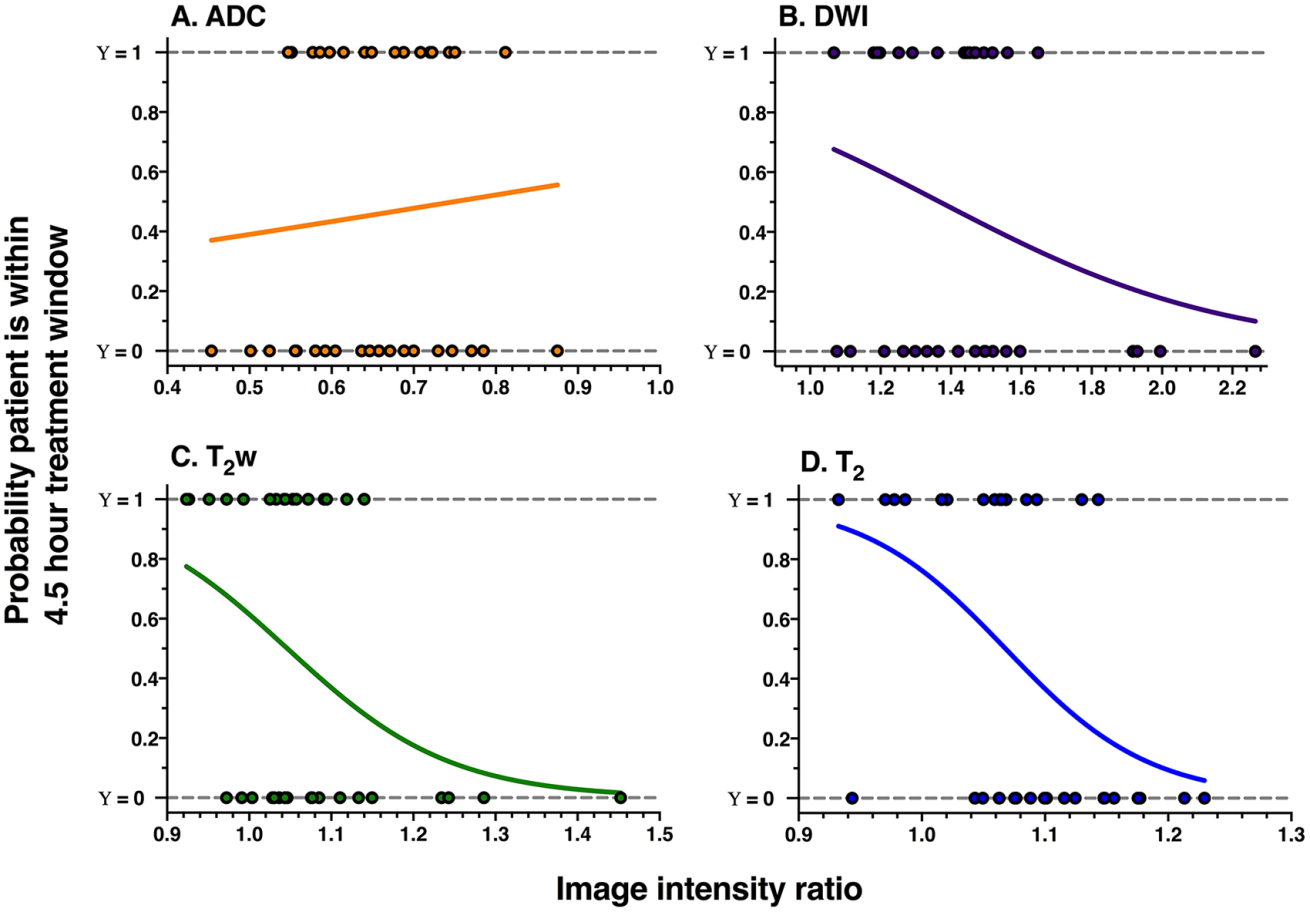
Probability plots for (A) ADC, (B) DWI, (C) T_2_w, and (D) T_2_ relaxation time image intensity ratios, derived from all 35 patients. The curves are the logistic fits that model the probability of a patient being within the 4.5-hour thrombolysis treatment window (y-axis) as a function of the image intensity ratio (x-axis). Dots represent image intensity ratios of individual patients who are within (Υ = 1) or beyond (Υ = 0) the treatment window. ADC indicates apparent diffusion coefficient; DWI, diffusion-weighted imaging.

Derived from logistic regressions, Figure 5 depicts that the probability that a patient is within the treatment window is higher when the DWI, T_2_w, and T_2_ ratios are smaller and that the T_2_ relaxation time ratio offers the highest probability (up to 90%). As the ADC values decrease during ischemia, higher ADC ratios indicate a higher probability of being within the treatment window. However, of those plots displayed in Figure 5, the results of the regressions indicate that only the T_2_ relaxation time and the T_2_w ratios were significant predictors of whether the patient was within the treatment window (Table 3). Table 3 also shows that the combined model involving all 4 image intensity ratios significantly predicted the probability of a patient being within the treatment window, but not to the same extent as the single-parameter regressions on the T_2_w ratio and, in particular, the T_2_ relaxation time ratio. Closer examination of the contribution of each parameter in the combined model (*P* values, Table 3) shows that the intercept was the only significant contributor to the model and that the T_2_ ratio, although not significant, had the most dominant contribution (*P* = .068). Overall, the T_2_ relaxation-time-ratio-only model was the best predictor of whether a patient is within or beyond the treatment window, as compared with other models, and it was the most significant and had the lowest AIC_c_.

**Table 3.** Information for the logistic regression analysis.

Model	β (SE)	*t*-statistic	*P*	Overall model evaluation			
				χ^2^	*df*	*P*	AIC_c_
ADC				0.24	33	.626	52.03
ADC ratio	1.79 (3.68)	0.49	.627	–	–	–	–
Intercept	–1.34 (2.43)	–0.55	.582	–	–	–	–
DWI				2.76	33	.097	49.50
DWI ratio	–2.44 (1.61)	–1.51	.130	–	–	–	–
Intercept	3.34 (2.33)	1.44	.150	–	–	–	–
T_2_w	–	–	–	5.38	33	.020*	46.88
T_2_w ratio	–10.05 (5.29)	–1.90	.057	–	–	–	–
Intercept	10.514 (5.58)	1.88	.060	–	–	–	–
T_2_				8.41	33	.004*	43.85
T_2_ ratio	–17.13 (7.09)	–2.42	.016*	–	–	–	–
Intercept	18.3 (7.64)	2.39	.017*	–	–	–	–
Combined				9.75	30	.045*	48.52
Intercept	22.05 (10.23)	2.15	.031*	–	–	–	–
ADC ratio	–0.07 (5.90)	–0.01	.990	–	–	–	–
DWI ratio	0.72 (3.52)	0.21	.837	–	–	–	–
T_2_w ratio	–7.57 (8.78)	–0.86	.389	–	–	–	–
T_2_ ratio	–14.05 (7.69)	–1.83	.068	–	–	–	–

Abbreviations: ADC, apparent diffusion coefficient; AICc, Akaike information criterion corrected for sample size; *df*, degrees of freedom, DWI, diffusion-weighted.

β is the estimated coefficient of the ratio or intercept, SE is the estimated standard error of β, *t*-statistic = β divided by the SE, *P* is the significance level.

* *P* < .05.

### Subcohort with FLAIR MRI

**Figure 6.**
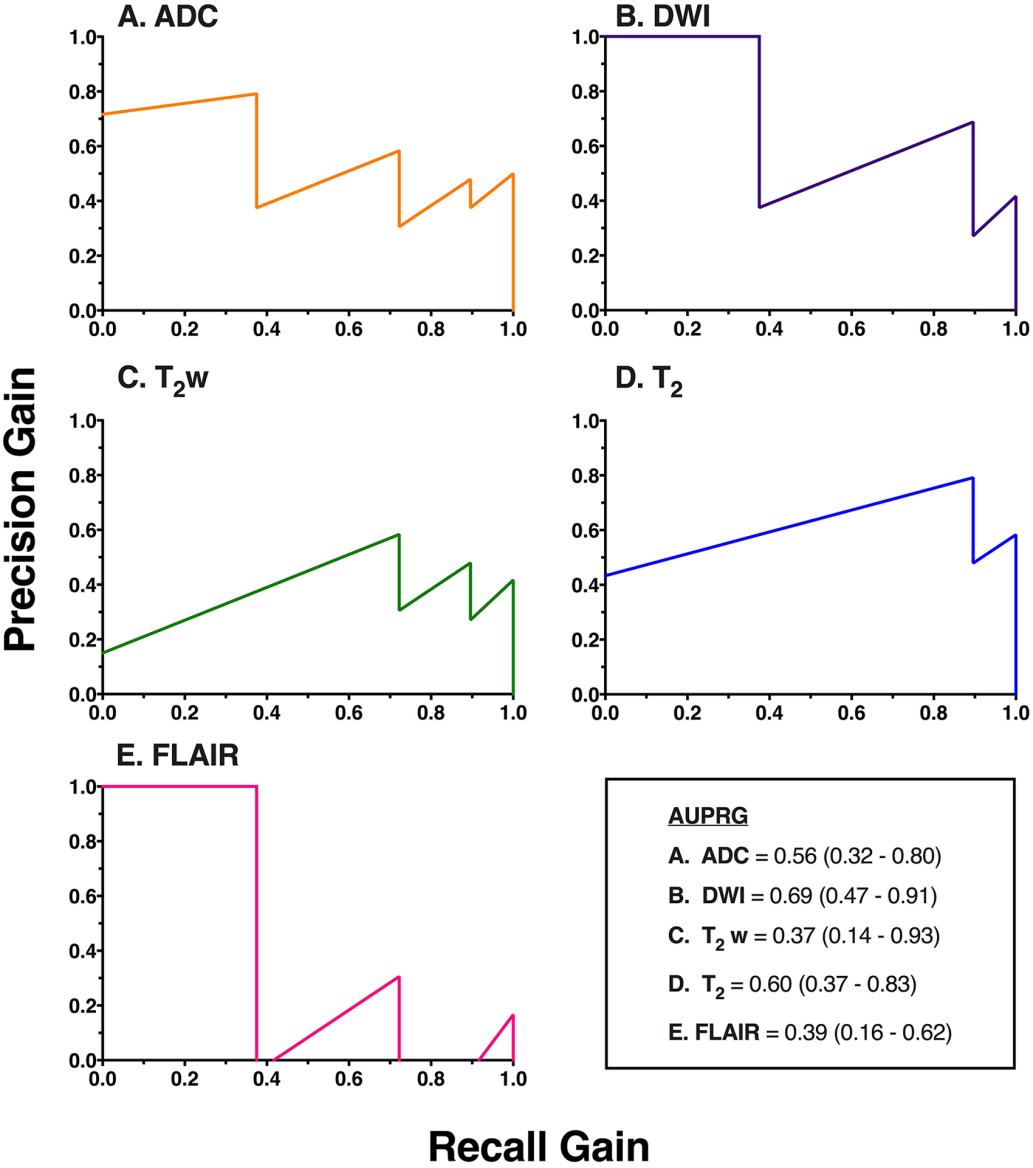
PRG curves and AUPRG curves from the subcohort of patients with additional FLAIR scans. The y-axis shows precision-gain, and the x-axis shows recall-gain values. Lines closest to the top right of the graph indicate parameters with high overall ability to identify patients scanned before 4.5 hours. An AUPRG of 0 indicates a trivial classifier, and positive and negative AUPRGs indicate more and less optimal classifiers, respectively. AUPRG values are given, with 95% confidence intervals in brackets. ADC, apparent diffusion coefficient; AUPRG, areas under the PRG; DWI, diffusion-weighted imaging; PRG, precision-recall-gain.

The agreement between raters of the DWI/FLAIR mismatch was intermediate to good, with a free-marginal Fleiss kappa value of 0.59 (CI = 0.36-0.82), and 72.8% agreement.^35,36^ All image intensity ratios did not correlate significantly with time from symptom onset (see SI Table 1 in supplementary materials), likely due to the small number of patients in the subcohort (Table 2). Figure 6 shows the PRG curves and associated AUPRGs. The AUPRGs were highest for the T_2_ relaxation time ratio and DWI ratio, suggesting both parameters have good overall ability at identifying patients within the thrombolysis treatment window. However, the T_2_ relaxation time curve was closest to the top right-hand corner of the graph, possibly suggesting a superior ability to the T_2_w ratio. The AUPRGs were lowest for the T_2_w and FLAIR ratios, demonstrating poor overall ability. Superior performance of T_2_ relaxation compared with other parameters was also reflected by the F_1_ score which was highest for the T_2_ relaxation time ratio (0.73, CI = 0.52-0.94), lower for the ADC (0.59, CI = 0.35-0.82), DWI (0.67, CI = 0.44-0.89), T_2_w (0.59, CI = 0.35-0.82), and FLAIR (0.57 CI = 0.34-0.81) ratios, and very low for the DWI/FLAIR mismatch (0.25, CI = 0.15-0.35).

## Discussion

In this study, the overall performance of quantitative and visual MRI-based methods for estimating stroke onset time, in the same cohort of hyperacute stroke patients, was compared. The T_2_ relaxation time ratio outperformed ADC, DWI, T_2_w, FLAIR image intensity ratios and the visual DWI/FLAIR mismatch, and combining ADC, DWI, T_2_w and T_2_ ratios showed no benefit. Results, therefore, suggest that the acquisition of T_2_ relaxation times, with ADC for lesion localization, may be sufficient for estimating stroke onset time.

Our results support recent conclusions drawn from preclinical^14,25,47,48^ and clinical^16,18^ studies that the T_2_ relaxation time detects brain ischemia and estimates onset time more accurately than MRI parameters derived from weighted images. Studies in rat models of ischemia reported strong relationships of the T_2_ relaxation with time from stroke onset^14,15,47^ and that onset time estimates made with T_2_ had a lower margin of error than when intensities of weighted images were used.^25,47,48^ The T_2_ relaxation time also demonstrated higher overall ability at differentiating between scans performed before and after 3 hours from ischemia onset in rat models.^25^

Patient studies have shown the T_2_ relaxation time to have a strong linear relationship with time from symptom onset^16-18^ and high overall ability at distinguishing between patients within and beyond the thrombolysis time window.^17,19^ In this study, the AUC of the T_2_ relaxation time ratio shows that 77% of the time, a randomly selected patient scanned within 4.5 hours of symptom onset had a lower T_2_ than a randomly selected patient scanned at a time later than 4.5 hours. Similarly, previous patient studies have reported 76% using hemispheric differences in T_2_ relaxation times at 1.5T for a 3-hour time window^17^ and at 3T for a 4.5-hour time window, 71% using the T_2_ change (difference), and 81% using a user-independent technique to quantify T_2_ changes.^19^

The potential utility of FLAIR imaging for estimating onset time has received considerable attention within the stroke imaging literature (see Etherton et al^8^ for review) but has only recently been directly compared with the T_2_ relaxation time.^16^ Results by Duchaussoy et al^16^ and from our study suggest that the T_2_ relaxation time will provide a more accurate estimation of stroke onset time than FLAIR-based approaches. Duchaussoy et al^16^ reported a stronger relationship for T_2_ relaxation with time from symptom onset compared with FLAIR image intensities (T_2_: *r* = 0.65 vs FLAIR: *r* = 0.18) in a cohort of stroke patients scanned within 4.5 hours of symptom onset. We extended these findings by showing that the stronger relationship with time also applies when scans performed up to 9 hours from symptom onset are considered, all patients: *r* = 0.49 (T_2_); subcohort: *r* = 0.35 (T_2_), *r_s_* = 0.25 (FLAIR). In further support, in the subcohort of patients with additional FLAIR scans, the T_2_ relaxation time ratio demonstrated a much higher overall ability than the FLAIR ratio at identifying patients scanned within the thrombolysis treatment window. The ability of the FLAIR ratio and the DWI/FLAIR mismatch approach was comparatively weak, which is in accordance with previous studies that have reported low sensitivity of FLAIR-based methods.^8,49,50^

The changes in T_2_ relaxation that occur during early ischemia reflect the radical changes in water dynamics caused by anoxic depolarization, whereby water is shifted from the extracellular to intracellular compartment, ie, cytotoxic edema, followed by the time-dependent breakdown of intracellular macromolecules.^51^ The same pathophysiological factors (cytotoxic edema with the breakdown of intracellular macromolecules) that cause changes in T_2_ relaxation, therefore, also contribute to the signal of images with a T_2_w component. This contribution will explain why other patient studies^23,24,28^ have reported a relationship for DWI and FLAIR image intensities with time from symptom onset. Despite the complexity of contributions to the measured T_2_ relaxation time, being a single quantitative parameter, it is a more accurate measure of stroke onset time (and, possibly, of pathophysiological changes in ischemia) than image intensities from weighted images which are also influenced by other factors such as proton-density, T_1_ relaxation, pulse sequence parameters, and inhomogeneities in B_0_ and B_1_. The increase in T_1_ relaxation during ischemia would decrease the T_2_w and FLAIR signals in lesions unless the images are acquired with very long time of repetition (TR) (>10 000 ms at 3T),^14^ which may explain why T_2_w and FLAIR ratios did not increase significantly in this study. Bias field problems may also be problematic in weighted images. The benefit of quantifying the T_2_ relaxation time is that in computing the T_2_ images, sources of error described above are removed. Thus, using T_2_ relaxation instead of weighted images for stroke timing uses the dependence of T_2_ on ischemia while removing confounding factors, making it a potentially more reliable stroke timer.

This study has some limitations that require consideration. First, it was a pilot study with a modestly sized unselected cohort of people with stroke symptoms, consisting of strokes of varying size and severity, that occurred in different vascular territories and tissue types. Recent results suggest the time dependency of T_2_ during the first 9 hours of stroke does not differ between grey and white matter,^18^ but it is currently unclear whether this is true for different vascular territories. A previous study^27^ found lesion size to be a mediating factor in the relationship of DWI image intensity ratios with time from onset. It is, therefore, possible that the wide range of lesion sizes included in this study introduced some variability in the data, which could underlie the weak correlations of some of the parameters. Other clinical variables, which could not be accounted for in this study, may also affect the relationship of image intensity ratios over time. For example, collateral status has been shown to influence the relationship of FLAIR image intensities with time from symptom onset.^52^

Second, due to the clinical nature of the study, most MRI scans were acquired after thrombolysis was administered (74.3% all patients and 82.4% in subcohort), which is not representative of patients with an unknown onset time. Nevertheless, based on both preclinical^53^ and clinical data,^18,54^ we do not think that rtPA will have had a significant effect on the T_2_ values within the lesion because the average ADC lesion values of the thrombolysed patients were not significantly different to the low ADC values of non-thrombolysed patients. Preclinical data suggest that if the ADC remains low following reperfusion, T_2_ continues to increase with time.^53^ In addition, a recent patient study^54^ showed rtPA does not alter net water uptake in the ischemic lesion, and so by extension, T_2_, which also reflects net water uptake, should not be altered. Furthermore, in our recent analysis of the patient cohort studied here, we found that the size of ADC lesions increased in 11 of 19 thrombolysed patients between initial (<9 hours onset) and follow-up scans (24+ hours), suggesting that ADC remained low long after rtPA, and thus rtPA would have had minimal or no effect on T_2_ in the lesion.^18^ However, to truly support this point, more information about blood flow would be needed, and a future study, including patients who undergo MRI before reperfusion therapies, is warranted. Regarding thrombolysis before MRI examination, it is well known in clinical practice that rtPA administration may make the previously ischemic lesion DWI/ADC negative.^55^ Potential normalization of DWI/ADC would not affect our analyses of the T_2_ MRI signal characteristics, because only lesions with ischemic ADC were included.

Given the above limitations, further investigation in a larger patient cohort is required. Such a study would enable more FLAIR scans so that the FLAIR image intensity ratio and DWI/FLAIR mismatch can be compared fully with other parameters. Additional scans should be acquired that offer information on collateral status to determine the effects of thrombolysis, and whether it mediates the relationship of other MR parameters with time from onset. A more extensive study would also allow separation of data analysis into different vascular territories and lesion sizes and should involve validation of results in an unseen test data set.

In conclusion, this study suggests that the T_2_ relaxation time may be the most accurate and reliable measure for estimating stroke onset time and that relying on weighted images as a method for stroke timing may be problematic. It also suggests that quantifying ADC for lesion identification and T_2_ for onset time estimation will be sufficient, and other parameters are not needed. In terms of clinical practice and feasibility, quantifying ADC (for lesion localization) and T_2_ relaxation times for onset time estimation requires minimal image processing. The MRI protocol adopted here is based on commonly available pulse sequences provided by all MRI vendors. Diffusion and multi-echo T_2_ are quick and easy to acquire (~5 minutes maximum, in total, in this study), and most scanners automatically produce ADC and T_2_ relaxation time images. With the advent of magnetic resonance fingerprinting^56^ under-sampled image acquisition of both parameters could result in even shorter acquisition times, which is imperative in emergencies such as ischemic stroke where “time is brain.”^57^ The clinical feasibility of using magnetic resonance fingerprinting for assessment of hyperacute stroke patients has recently been demonstrated, where 2DT_1_ and FLAIR images and T_1_ and T_2_ relaxation time maps were acquired simultaneously within 4 minutes and 24 seconds.^16^ The post-processing steps used in this paper were exploratory and not designed for a clinical setting. However fast and automatic delineation of ischemic regions on ADC images is already widely available,^58^ and we have recently proposed a user-independent method for quantifying the impact of stroke on the human brain which has the potential to be easily automated for a clinical setting.^19^

## Supplemental Material

Supplementary_Materials_3 – Supplemental material for A Comparison of T2 Relaxation-Based MRI Stroke Timing Methods in Hyperacute Ischemic Stroke Patients: A Pilot Study Click here for additional data file. Supplemental material, Supplementary_Materials_3 for A Comparison of T2 Relaxation-Based MRI Stroke Timing Methods in Hyperacute Ischemic Stroke Patients: A Pilot Study by Bryony L McGarry, Robin A Damion, Isabel Chew, Michael J Knight, George WJ Harston, Davide Carone, Peter Jezzard, Amith Sitaram, Keith W Muir, Philip Clatworthy and Risto A Kauppinen in Journal of Central Nervous System Disease
