# Supplementary material for: A Comparison of T2 Relaxation-Based MRI Stroke Timing Methods in Hyperacute Ischemic Stroke Patients: A Pilot Study
Source: J Cent Nerv Syst Dis. 2020 Sep 12;12:1179573520943314. doi: 10.1177/1179573520943314 (PMC7488882; doi:10.1177/1179573520943314)
Supplement: Supplementary_Materials_3 – Supplemental material for A Comparison of T2 Relaxation-Based MRI Stroke Timing Methods in Hyperacute Ischemic Stroke Patients: A Pilot Study [file Supplementary_Materials_3.pdf]

Supplementary Materials

A comparison of T_2 relaxation-based MRI stroke timing methods in hyperacute ischemic stroke patients: A pilot study

ITEM 1 – Description and definition of performance metrics used in the context of this study

Performance of MRI classifiers was assessed using measures that consider true positive (TP), false positive (FP), true negative (TN) and false negative (FN) rates, where TP and TN refer to the number of patients *correctly* identified as within or beyond the treatment window, respectively. FP and FN refer to the number of patients *incorrectly* identified as within or beyond the treatment window, respectively. The measures used, defined in the context of this study, are given below.[1]

Measures of accuracy

- **Accuracy:** the overall ability of the classifier to differentiate between patients within and beyond the treatment window correctly (TP+TN/TP+TN+FP+FN).
- **Sensitivity/recall:** the ratio of patients correctly identified as within the treatment window to all the patients that were within the treatment window (TP/TP+FN). Represents the ability of the classifier to identify patients within the treatment window correctly.
- **Specificity:** the ratio of patients correctly identified as beyond the treatment window to all the patients that were beyond the treatment window (TN/TN+FP). Represents the ability of the classifier to identify patients beyond the treatment window correctly.

Measures of correctness

- **Precision / positive predictive value (PPV):** the ratio of patients correctly predicted to be within the treatment window to the total predicted to be within the treatment window (TP/TP+FP). Approximates the probability that a patient with an image intensity ratio below the optimal cut-off (or above for ADC) is within the treatment window.
- **Negative predictive value (NPV):** the ratio of patients correctly predicted to be beyond the treatment window to the total predicted to be beyond the treatment window (TN/TN+FN). Approximates the probability that a patient with an image intensity ratio above the optimal cut-off (or below for ADC) is beyond the treatment window.

Performance metrics used for all 35 patients

- **Receiver operating characteristic (ROC) curves** demonstrate the trade-off between sensitivity and specificity for every possible image intensity ratio cut-off. Classifiers with high discriminative ability have lines closest to the top left-hand corner of the ROC plot, and

classifiers with random performance are close to the 0.5-area reference line, which is a straight diagonal line from (0,0) to (1,1).[2]

- **The area under the ROC (AUC)** is a numerical indication of overall performance. An AUC of 0.5 indicates a classifier that performs no better than random guessing and an AUC of 1 indicates a perfect classifier.[3] In the context of this study, the AUC is an estimate of the probability that a randomly selected scan before 4.5 hours will have an image intensity ratio lower (or higher for ADC) than the image intensity ratio of a randomly selected scan performed after 4.5 hours.
- **The Youden J Index** (max [sensitivity + specificity – 1]) identifies the cut-off that minimizes misclassification by giving equal weight to sensitivity and specificity and corresponds to the point on the ROC curve with a vertical distance furthest from the 0.5 reference line, where the summation of sensitivity and specificity are maximum.[4,5] The image intensity ratio with the highest Youden J index will, therefore, be the one that can be used to distinguish the highest number of patients within and beyond the treatment window.
- **Logistic Regression** is used to describe and test hypotheses about the relationship between a categorical outcome variable, Y (e.g., within or beyond the treatment window) and one or more independent predictor variables (e.g., image intensity ratios).[6] The logistic model predicts the natural logarithm, Y, of the ratio of the probability of an event happening (e.g., the patient is within the treatment window) to the probability of the event not happening (e.g., the patient is beyond the treatment window). The logistic model, therefore, takes the form of:

$$Y = \alpha + \beta_1\chi_1 + \beta_2\chi_2 + \beta_3\chi_3 + \beta_4\chi_4 \ldots. \quad \text{(SI 1)}$$

In the context of this study, Y is the predicted log-odds of the patient being within the treatment window ($Y = 1$ for onset time < 270 minutes, $Y = 0$ for onset time > 270 minutes) α is the estimated intercept, β_1 is the estimated regression coefficient and $\chi_{1\ldots n}$ are the predictor variables (e.g. image intensity ratio of ADC, DWI, T_2w, T_2). The probability of being within the treatment window (P) is therefore calculated as:

$$\frac{e^{(\alpha + \beta_1\chi_1 + \beta_2\chi_2 + \beta_3\chi_3 + \beta_4\chi_4 \ldots.)}}{1 + e^{(\alpha + \beta_1\chi_1 + \beta_2\chi_2 + \beta_3\chi_3 + \beta_4\chi_4 \ldots.)}} \quad \text{(SI 2)}$$

- **Akaike information criteria (AIC)**[7] is a measure of the quality of a model relative to other models in the same dataset and represents the amount of information lost by using the model.[8] The most accurate model has the lowest AIC.[7] The AIC is calculated using the number of fitted parameters, including the intercept in the model (k), and the maximum likelihood estimate for the model (L):

$$AIC = -2\ln(L) + 2k. \quad \text{(SI 3)}$$

However, when k and the sample size (n) is less than 40, AIC corrected for sample size (AIC_c) is recommended[8] and was thus used in this study. Where:

$$AIC_c = AIC + \frac{2k(k+1)}{n-k-1} \quad \text{(SI 4)}$$

The lowest AIC the generalized linear model produced by the logistic regression analysis with the lowest AIC_c value represents the one that, in comparison to other models, best predicts whether a patient is within the thrombolysis treatment window.

Performance metrics used for sub-cohort with FLAIR MRI

- **Precision-recall-gain (PRG) methods** developed by Flach and Kull[9] is a more interpretable variation of the traditional precision-recall analysis. Precision-gain and recall-gain are linearized versions of precision (or PPV) and recall (or sensitivity) values. PRG methods have been shown to be an appropriate alternative to ROC analysis in class imbalanced data sets and overcome problems associated with traditional precision-recall analysis.[9]
- **The precision-recall-gain (PRG) curve** provides a visual indication of overall performance by plotting precision-gain on the y-axis and recall-gain on the x-axis, ignoring negative gains. Lines closest to the top right of the graph indicate parameters with high overall ability to identify patients scanned before 4.5 hours.[9]
- **The area under the PRG curve (AUPRG)** provides a numerical indication of overall performance. An AUPRG of zero indicates a trivial classifier and positive and negative AUPRGs indicate more and less optimal classifiers, respectively.[9] There is no predefined limit as to what constitutes a high overall performance, but a higher AUPRG would indicate a classifier that performs better at identifying patients within the treatment window.

The F_1 score[10] is considered an appropriate measure for evaluation of a class-imbalanced data set, where the higher the F_1 score, the better the overall performance.[1] The F_1 score gives equal weighting to the importance of precision and recall and is defined as the harmonic mean of precision and recall:

$$F_1 = 2\left(\frac{precision \times recall}{precision + recall}\right) \quad \text{(SI 5)}$$

SI Table 1. Test statistics for Shapiro-Wilk and correlation analyses.

Classifier	Shapiro-Wilk		Correlation Coefficient					
	W	*p*	Pearson's r (r)	*p*	Spearman's rank (r_s)	*p*	Kendall's Tau (r_t)	*p*
**All 35 patients**								
ADC	0.99	0.998	- 0.07	0.708	- .09	0.620	- 0.08	0.514
DWI†	0.90	0.005*	0.25	0.153	0.15	0.395	0.11	0.370
T_2w†	0.86	0.001*	0.36	0.032*	0.22	0.201	0.17	0.168
T_2	0.98	0.779	0.49	0.003*	0.48	0.005*	0.32	0.006*
**Sub-cohort with FLAIR**								
ADC	0.99	0.999	- 0.17	0.511	-.10	0.715	- 0.09	0.621
DWI	0.90	0.065	0.39	0.119	0.27	0.305	0.18	0.323
T_2w	0.91	0.090	0.36	0.152	0.28	0.286	0.22	0.217
T_2	0.96	0.725	0.35	0.166	0.39	0.127	0.29	0.099
FLAIR†	0.89	0.044*	0.56	0.831	0.25	0.342	0.21	0.266

Note. W = Shapiro-Wilk statistic, *p* = significance level, * indicates significant relationship as $p < .05$, †indicates a parameter that was not normally distributed and therefore Spearman's Rank and Kendall's Tau results should be referred to. r = Pearson's r correlation coefficient for normally distributed data, r_s = Spearman's rank correlation coefficient for non-normally distributed data, and r_t is the Kendall's Tau correlation coefficient for non-normally distributed data.

SI Table 2. Statistics for comparisons of areas under the ROC curves.

Parameters	Difference between AUCs (SE)	95% CI	z-statistic	*p*
**ADC vs DWI**	0.09 (0.09)	-0.08 – 0.26	1.06	0.288
**ADC vs T_2w**	0.12 (0.14)	-0.15 – 0.38	0.84	0.399
**ADC vs T_2**	0.22 (0.12)	0.00 – 0.45	1.92	0.055
**DWI vs T_2w**	0.02 (0.09)	-0.15 – 0.20	0.26	0.794
**DWI vs T_2**	0.13 (0.11)	-0.08 – 0.34	1.24	0.215
**T_2w vs T_2**	0.11 (0.12)	-0.10 – 0.32	1.01	0.313

Note. AUC = area under the receiver operating characteristic (ROC) curve, SE = standard error, CI = confidence interval, *p* = significance level.

References

1. Bekkar M, Djemaa DHK. Evaluation measures for models assessment over imbalanced data aets. *J Inf Eng Appl.* 2013;3 (10):27 -38.

2. Zweig MH, Campbell G. Receiver-operating characteristic (ROC) plots: a fundamental evaluation tool in clinical medicine. *Clin Chem*. 1993;39(4):561-577.

3. Hanley JA, McNeil BJ. A method of comparing the areas under receiver operating characteristic curves derived from the same cases. *Radiology*. 1983;148(3):839-843.

4. Habibzadeh F, Habibzadeh P, Yadollahie M. On determining the most appropriate test cut-off value: the case of tests with continuous results. *Biochem Medica*. 2016:297-307.

5. Youden WJ. Index for rating diagnostic tests. *Cancer*. 1950;3(1):32-35.

6. Peng CYJ, Lee KL, Ingersoll GM. An introduction to logistic regression analysis and reporting. *J Educ Res*. 2002;96(1):3-14.

7. Akaike H. A new look at the statistical model identification. *IEEE Trans Autom Control.* 1974;19(6):716-723.

8. Symonds MRE, Moussalli A. A brief guide to model selection, multimodel inference and model averaging in behavioural ecology using Akaike's information criterion. *Behav Ecol Sociobiol.* 2011;65(1):13-21.

9. Flach P, Kull M. Precision-Recall-Gain curves: PR analysis done right. In: Cortes C, Lawrence ND, Lee DD, Sugiyama M, Garnett R, eds. *Advances in Neural Information Processing Systems 28*. Curran Associates, Inc.; 2015:838–846.

10. Rijsbergen CJV. *Information Retrieval.* 2nd ed. Newton, MA, USA: Butterworth-Heinemann; 1979.
